# Neuropeptide Y in the amygdala contributes to neuropathic pain-like behaviors in rats *via* the neuropeptide Y receptor type 2/mitogen-activated protein kinase axis

**DOI:** 10.1080/21655979.2022.2051783

**Published:** 2022-03-21

**Authors:** Wenhui Yan, Wuchao Liu, Junlu Wu, Lipei Wu, Shihai Xuan, Weiwei Wang, Anquan Shang

**Affiliations:** a Department of Laboratory Medicine Yangzhi Rehabilitation Hospital (Shanghai Sunshine Rehabilitation Center), Tongji University School of Medicine, Shanghai, P.R. China; b Department of Laboratory Medicine, Tinghu People’s Hospital, Yancheng, P.R. China; c Department of Neurorehabilitation, Yangzhi Rehabilitation Hospital (Shanghai Sunshine Rehabilitation Center), Tongji University School of Medicine, Shanghai, P.R. China; d Department of Laboratory Medicine, Shanghai Tongji Hospital, Tongji University School of Medicine, Shanghai, P.R. China; e Department of Laboratory Medicine, Dongtai People’s Hospital & Dongtai Hospital of Nantong University, Yancheng, P.R. China; f Department of Pathology, Tinghu People’s Hospital, Yancheng, P.R. China

**Keywords:** Neuropeptide Y, NPY2R, MAPK signaling pathway, amygdala, neuropathic pain, mechanical withdrawal threshold, thermal withdrawal latency

## Abstract

Neuropeptide Y (NPY) is a highly conserved endogenous peptide in the central and peripheral nervous systems, which has been implicated in nociceptive signaling in neuropathic pain. However, downstream mechanistic actions remain uncharacterized. In this study, we sought to investigate the mechanism of NPY and its receptor NPY2R in the amygdala in rats with neuropathic pain-like behaviors induced by chronic constriction injury (CCI) of the sciatic nerve. The expression of NPY and NPY2R was found to be aberrantly up-regulated in neuropathic pain-related microarray dataset. Further, NPY was found to act on NPY2R in the basolateral amygdala (BLA). As reflected by the decrease in mechanical withdrawal threshold (MWT) and thermal withdrawal latency (TWL) as well as the increase of NPY expression in the amygdala of rats with neuropathic pain-like behaviors, NPY was closely related to the effect of amygdala nerve activity in neuropathic pain. Subsequently, mechanistic investigations indicated that NPY2R activated the MAPK signaling pathway in the amygdala. NPY2R-induced decrease of MWT and TWL were also restored in the presence of MAPK signaling pathway antagonist. Moreover, it was revealed that NPY2R overexpression promoted the viability while inhibiting the apoptosis of microglia. Taken together, NPY in the amygdala interacts with NPY2R to activate the MAPK signaling pathway, thereby promoting the occurrence of neuropathic pain.

## Graphical Abstract

**Figure:**
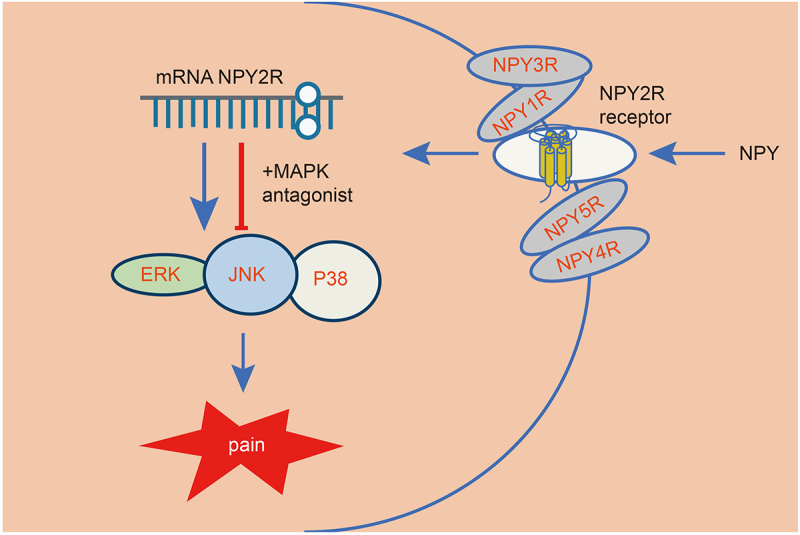

## Background

Neuropathic pain is generated by injury or dysfunction of the somatosensory nervous system and may be highly disabling [1]. It is estimated that 8% of the population suffer from neuropathic pain [2]. Neuropathic pain is diagnosed through history and neurological examination [3]. The neuropathic pain is accompanied by spontaneous and stimulus-evoked pain sensations, and the underlying maladaptive mechanisms include peripheral sensitization of pain, aberrant afferent neuron excitability, as well as central sensitization [4]. At present, the first-line pharmacotherapy for neuropathic pain mainly includes the use of antidepressants and anticonvulsants, which, unfortunately, exhibits limited efficacy [5]. Moreover, it is believed that microglia play an important role in the pathogenesis of neuropathic pain [6], with signaling between microglia and neurons a critical player in neuropathic pain transmission [7], but the underlying mechanism remains to be established.

It has been reported that amygdala had a role to confer in both acute and chronic pain and is associated with negative emotions [8]. A previous study has suggested that neuropeptide Y (NPY) was abundantly expressed in the amygdala [9]. NPY is regarded as a sympathetic neurotransmitter that acts through its various receptors (Y1-Y5R) [10]. Of note, it has been revealed that NPY is involved in neuropathic pain, which serves as a potential therapeutic target [11]. Furthermore, the role of NPY and its receptor Y2R (NPY2R) has been highlighted for its involvement in the spinal regulation of neuropathic pain [12].

The mitogen-activated protein kinase (MAPK) signaling pathway is considered to be a canonical signaling pathway for a variety of receptor tyrosine kinases [13]. As previously reported, activated NPY2R could induce lipid accumulation in murine adipocytes, which may be associated with the regulation of the MAPK signaling pathway [14]. Moreover, increased plasma concentration of NPY triggers cardiac dysfunction and hypertrophy by modulating the MAPK signaling pathway [15]. Intriguingly, the suppression of the TAK1-p38 MAPK/NF-κB signaling pathway in RAW264.7 and RSC96 Schwann cells could alleviate chronic constriction injury (CCI) of sciatic nerve-induced neuropathic pain-like behaviors [16]. Additionally, p38-MAPK inhibited by downregulated microRNA-155 contributes to ameliorated mechanical allodynia as well as thermal hyperalgesia in bortezomib rats, which alleviates neuropathic pain-like behaviors in the course of chemotherapeutic bortezomib [17].

Taking all the aforementioned findings into consideration, we then hypothesized that NPY in amygdala may interact with its receptor NPY2R and regulate the MAPK signaling pathway, thereby affecting neuropathic pain. Thus, the purpose of the current study was to explore the roles and downstream mechanisms of NPY in the amygdala contributing to neuropathic pain.

## Materials and methods

### Ethical approval

This study was approved by the ethics committee of Shanghai Tongji Hospital (No. 2021-DW-SB-108) and all animal experimental procedures were performed following the *Guide for the Care and Use of Laboratory Animals* issued by the National Institutes of Health.

### Microarray-based gene expression profiling

The neuropathological pain-related gene expression dataset GSE30691 was retrieved from the Gene Expression Omnibus (GEO) database. This dataset contained L4-5 dorsal root ganglia samples from 12 control rats and 3 rats with CCI of sciatic nerve. Differentially expressed genes in CCI rats were screened by differential expression analysis using the GEO2R online tool (|logFC|> 1 and *p* value <0.05).

### Experimental animals

A total of 160 8-week-old Sprague-Dawley (SD) male rats, weighing 200–260 g, were randomly divided into 8 groups (*n* = 20): sham group (sham-operated rats), CCI group (untreated CCI rats), BLA group (CCI rats with basolateral amygdala [BLA] damage), ODN group (CCI rats treated with NPY2R antisense oligonucleotide [ODN]), NPY2R group (CCI rats treated with NPY2R agonist), and ERK group (CCI rats treated with extracellular signal-regulated kinase [ERK] antagonist SCH772984), JNK group (CCI rats treated with jun N-terminal kinase [JNK] antagonist SP600125), and p38 group (CCI rats treated with p38 MAPK antagonist B203580).

Rats to be modeled were placed in a stereotactic frame to expose the sciatic nerve. The sciatic nerve was exposed through blunt dissection in the middle thigh where the bifurcation of the femoris nerve system through the anterior biceps was located. Under the anatomical microscope, a 4.0 suture was used to gently tie 4 places with an interval of 1 mm. When a small muscle twitch of the left hind limb was observed, the incision was sutured layer by layer, and the rats were housed in separate cages (a rat/per cage) [18,19].

In the sham-operated rats, only the sciatic nerve trunk was exposed and the wound was sutured.

Rats subjected to BLA + CCI were anesthetized with pentobarbital sodium (3%). The rats were placed in the stereotactic frame (David Kopf instrument) and the bipolar electrode was implanted into the right BLA (position: anteroposterior distance: –2.8 mm, medial lateral: –4.8 mm; dorsal abdomen, 8.0 mm from dura mater). The electrodes were fixed on the skull with dental cement and three stainless steel screws, one of which was used as the ground electrode. The rats were given buprenorphine and recovered from the operation one week before the kindling operation [20,21].

On the 7^th^ day after the operation, pentobarbital sodium was used to anesthetize the rats (3%, mg/kg, which could be increased if necessary). A 30-gauge puncture needle was used to puncture through the gap L5 and L6. The puncture was successful if the tail of the rat showed rapid swing. Rats were intrathecally injected with 5 μg/μL NPY2R ODN (30 μL), 150 μg NPY2R agonist, or MAPK signaling pathway antagonists (150 μg ERK antagonist, 150 μg JNK antagonist, or 150 μg p38 MAPK antagonist). Those untreated CCI rats were injected with equal volume of normal saline.

Mechanical withdrawal threshold (MWT) and thermal withdrawal latency (TWL) were, respectively, measured 2 days before CCI (T0), and 1, 4, 14 and 21 days after the surgery (T), as well as 15 min, 1.5 h, 3.0 h, 4.5 h and 6.0 h after intrathecal administration (5 rats in each group were selected for analysis).

### Measurement of MWT and TWL

MWT was evaluated using von Frey filament. Each metatarsal surface was stimulated with each filament (2.0–26.0 g) 10 times, which started with 2 g filaments, and gradually increased until the rats responded to grasp the claws off the surface of the test device. The medial, ipsilateral and contralateral hindfoot areas were measured. Once the reaction was detected, the light filaments were used to estimate the sensory threshold of each claw in turn. The MWT was calculated based on the following formula: 50% MWT (g) = (10 ^ 255 [*Xf* + *kδ*])/10,000. To calculate the TWL, the outer sole of the rat foot was exposed to the hot plate (50°C). Initial withdrawal latency and duration were recorded. Each claw was stimulated three times every 10 min, and the average value was calculated [22,23].

### Synthesis of NPY2R antisense ODN sequence

According to the NPY2R gene (NPY2R; GeneID: 18,167) in the gene bank, the antisense ODN sequence of NPY2R gene was designed: NPY2R-ODN: 5’-TCTGCACCTAATGGGCCC-3’, which was diluted with normal saline before use [24].

### Reverse transcription-quantitative polymerase chain reaction (RT-qPCR)

The RNA was extracted from tissues and cells using Trizol reagent (15,596,026, Invitrogen, Car, Cal, USA). According to the instructions of PrimeScript RT reagent kit (RR047A, Takara, Kyoto, Japan), the RNA was reverse-transcribed into cDNA, and the synthesized cDNA was subjected to RT-qPCR determination using Fast SYBR Green PCR kit (Applied Biosystems, Carlsbad, CA, USA) in the ABI prism 7300 RT-PCR system (Applied Biosystems). Glyceraldehyde-3-phosphate dehydrogenase (GAPDH) served as the internal reference, and the relative mRNA expression of genes of interest was analyzed by 2^−ΔΔCT^ method. The primer sequences are shown in Supplementary Table 1 [25].

### Immunofluorescence staining

The amygdala of the rats was fixed overnight in Bouin solution. After dehydration, the samples were paraffin-embedded and longitudinally sectioned. Next, the sections were immersed in 3% methanol H_2_O_2_ for 20 min. Following antigen retrieval, the sections were cooled and sealed with a normal goat serum sealing solution (C-0005, Shanghai Haoran Biotechnology Co., Ltd., Shanghai, China) at room temperature for 20 min. The sections were incubated overnight at 4°C with anti-rabbit primary antibodies against NPY (ab234527, 1:2000; Abcam, Cambridge, UK), NPY1R (ab216966, 1:200; Abcam), NPY2R (PA5-77,517, 1:2000; Thermo Fisher Scientific). The sections were then incubated with the secondary antibody goat anti-rabbit against immunoglobulin G (IgG) H&L (Alexa Fluor® 488) (ab150077, 1:2000, Abcam) and then rested at 37°C for 1 h, followed by incubation with 4,6-diamidino-2-phenylin-dole (DAPI) for 5 min in the dark. Next, an anti-fading agent was used to seal the sections. Finally, the sections were observed and images were photographed under a fluorescence microscope [26].

### Immunohistochemistry

The steps before incubating the primary antibody were the same as those of immunofluorescence staining. The sections were incubated with primary antibody rabbit anti-rat against NPY (ab234527, 1:500; Abcam) overnight at 4°C and then with secondary antibody goat anti-rabbit against IgG (ab6785, 1:1000, Abcam) at 37°C for 20 min. Subsequently, a horseradish peroxidase-labeled Streptomyces ovalbumin working solution (0343–10,000 U, IMUNBIO, Beijing, China) was utilized to treat the sections at 37°C for 20 min. Following diaminobenzidine (DAB, ST033, Whiga, Guangzhou, China) color development, the sections were counterstained with hematoxylin (PT001, Bioon, Shanghai, China) for 1 min, treated with 1% ammonia water to revert to blue, dehydrated with gradient alcohol, and permeabilized with xylene. Finally, the sections were sealed with neutral resin, and observed under a microscope. Five fields of high-magnification view were randomly selected for each section, with 100 cells per field counted [27].

### Western blot assay

After rats were euthanized, the amygdala brain area was collected for extraction of the total protein for Western blot assay. The supernatant was obtained by centrifugation after 500 μL precooled 1 × lysis buffer w used to lyse the cells. The total protein sample, 30 pounds (mol/L), was separated by 10% sodium dodecyl sulfate polyacrylamide gel electrophoresis at 100 V and transferred to a polyvinylidene fluoride membrane. After sealing with tri-buffered saline Tween-20 (TBST) containing 5% skimmed milk for 1 h at room temperature, the membrane was incubated with diluted anti-rabbit antibodies against NPY (#11,976, 1:1000; Cell signaling technology, Danvers, MA, USA), NPY1R (ab91262,1:1000; Abcam), NPY2R (PA5-77,517, 1:200; Thermo Fisher Scientific), p38 MAPK (#8690, 1:2000, Cell Signaling Technology), ERK (#4370, 1:2000, Abcam) and GAPDH (ab9485, 1:5000; Abcam; internal inference) at 4°C overnight. Next, the membrane was incubated with horseradish peroxidase-labeled secondary antibody goat anti-rabbit against IgG (S0001, Affinity BioReagents Inc., Golden, Colorado, USA) for 1 h. The enhanced chemiluminescence kit (BB-3501, Amersham, Arlington Heights, IL, USA) was used for visualization of bands, which were quantified in the gel imager [28,29].

### Co-immunoprecipitation (Co-IP)

The rats were euthanized, and the total protein was extracted from excised amygdala tissues for Co-IP assay. The supernatant was obtained by centrifugation after tissue lysis using 500 μL pre-cooled 1 × lysis buffer at 4°C on ice. Next, 5 μL Agrose A/G beads and 5 μL (1 μg) antibodies were added to NPY and NPY2R protein samples, respectively. The total amounts of beads and antibodies used in each experiment were calculated. The antibodies and beads were mixed with a lysis buffer. The protein supernatant was added to the separated beads for immunoprecipitation for 3 h, and then the beads were washed with 1 × lysis buffer three times. Subsequently, 35 μL 1 × lysis buffer was added to the beads, followed by a mixture with an equal volume of 2 × sodium dodecyl sulfate sample buffer. After boiling for 10 min, the mixture was centrifuged, and 10 μL sample was loaded to polyacrylamide gel electrophoresis for Western blot assay [30].

### Cell culture and transfection

Mouse microglia (BV-2 cells) and rat astrocytes (C6 cells) were purchased from Procell Life Science & Technology Co., Ltd. (Wuhan, China) and mouse midbrain dopaminergic neurons (MN9D cells) were purchased from Tongpai (Shanghai) Biotechnology Co., Ltd. (Shanghai, China). These cell lines were incubated with Dulbecco’s modified Eagles Medium (DMEM)/F12 supplemented with 10% FCS (Gibco, Carlsbad, California, USA), 10% fetal bovine serum (FBS, Invitrogen), and penicillin streptomycin (Sigma-Aldrich, St Louis, MO, USA) at 37°C with 5% CO_2_. After surface-adherent growth, the cells were detached with 0.25% trypsin (Sigma-Aldrich), and the cells in logarithmic growth phase were harvested for subsequent experiments.

The overexpression vectors and shRNAs were synthesized by GenePharma (Shanghai, China). The cells were seeded in six-well plates at a density of 5 × 10^5^ cells/well. When the cell confluence reached 70%, the cells were transfected using Lipofectamine 3000 (Invitrogen) kits (L3000001; Thermo Fisher Scientific). Briefly, 2.5 μg target plasmid, 5 μL p3000^TM^ and 5 μL Lipofectamine 3000 were respectively diluted with 250 μL serum-free Opti-MEM (Gibco) medium, and allowed to stand for 15 min. The mixtures were added to the each well of the plate and cultured in a 5% CO_2_ incubator at 37°C. After 6 h, the medium was renewed with a complete medium for another 48-h incubation and the cells were collected to determine the transfection efficiency for subsequent experiments. Then, the cells were assigned into five groups, as shown in Supplementary Table 2.

### Cell counting kit-8 (CCK-8) assay

Cell viability was detected using cell counting kits by CCK-8 method (Dojindo, Kumamoto, Japan). BV-2 cells in logarithmic growth phase were seeded into a 96-well culture plate with the density of 1 × 10^3^ cells/well. The cells were transfected according to the aforementioned grouping with three repeated wells set for each group. After 0, 24, 48, 72 and 96 h of culture, 10 μL CCK-8 solution was added into each well, followed by incubation for 2 h. The optical density value was then determined at 450 nm using a microplate reader [31].

### 5-ethynyl-2-deoxyuridine (EdU) assay

Cell viability was tested with EdU Kits (KeyGen, Nanjing, China). EdU was labeled with kFuor488 and showed green fluorescence. The BV-2 cells were seeded into a 96-well plate at a density of 1 × 10^3^ cells/well. After transfection, the cells were incubated for 24 h, then incubated with 50 nm EdU solution for 2.5 h, and stained with Hoechst 33,342 for 30 min avoiding light exposure. Finally, EdU-positive cells were observed and counted under a fluorescence microscope. EdU-positive cell rate = EdU-positive cell number/the number of Hoechst33342-stained cells × 100% [32].

### Flow cytometry

Forty-eight hours after the transfection, the cells were detached with 0.25% ethylenediaminetetraacetate (EDTA)-free trypsin. The cells were centrifuged to discard the supernatant. According to the instructions of Annexin V-fluorescein isothiocyanate conjugate (FITC) cell apoptosis detection kit (556,547, Shuojia Biotechnology Co., Ltd., Shanghai, China), Annexin-V-FITC, propidium iodine (PI) and HEPES buffer solution were mixed into Annexin-V-FITC/PI staining solution at the ratio of 1:2:50. Next, per 100 μL of dye solution was used to resuspend 1 × 10^6^ cells. After the incubation at room temperature for 15 min, 1 mL of HEPES buffer solution was added to the mixture. The fluorescence of FITC and PI was measured by band-pass filters of 515 nm and 620 nm, respectively, with an excitation wavelength of 488 nm using flow cytometry [33].

### Statistical analysis

All data were analyzed using SPSS 21.0 statistical software (SPSS, IBM, Armonk, NY, USA). The measurement data were expressed by mean ± standard deviation. Data of two groups with normal distribution and homogeneous variance were compared by unpaired *t*-test, and those among multiple groups by one-way analysis of variance (ANOVA) with Tukey’s post hoc test. Data at different time points were compared with repeated measures of ANOVA. The difference was statistically significant at *p* < 0.05.

## Results

### Effect of amygdala nerve activity on neuropathic pain might be associated with NPY expression

**Figure 1.**
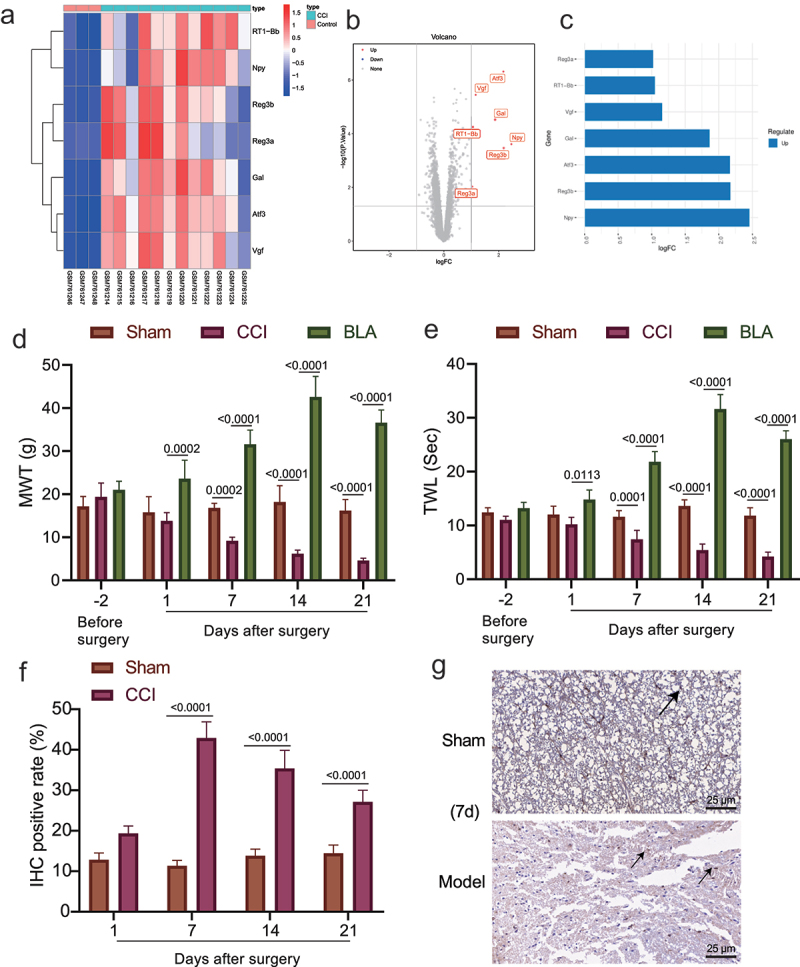
The role of amygdala nerve activity in rats with neuropathic pain-like behaviors is related to NPY expression. A-B, Heatmap (a) and volcano map (b) of differentially expressed genes between samples of control (*n* = 3) and CCI (*n* = 12) rats in the GSE30691 dataset. (c) The logFC values of differentially expressed genes between samples of control and CCI rats in the GSE30691 dataset. FC, fold change. (d) The MWT of CCI rats with/without BLA kindling at different time points. * *p* < 0.05 *vs*. untreated CCI rats. (e) The MWT of CCI rats at different time points. * *p* < 0.05 *vs*. untreated CCI rats. (f)-(g) Statistics (f) and NPY immunohistochemistry-positive staining (g) of sciatic nerve tissue of the CCI rats and the sham-operated rats on the 1^st^, 7^th^, 14^th^, and 21^st^ day after modeling (The arrow points to NPY positive cells). * *p* < 0.05 *vs*. sham-operated rats *n* = 5.

The lack of obvious clinical and pathological features makes neuropathic pain difficult to diagnose [34], and the mechanism leading to neuropathic pain remains to be established. First, the neuropathologic pain-related gene expression dataset GSE30691 was retrieved from the GEO database and differential expression analysis was performed using the GEO2R online tool based on the criteria of |logFC|> 1, *p* value < 0.05. Seven differentially expressed genes were identified in the samples of CCI rats, all being upregulated (Figure 1a and 1b). The largest fold change was found in NPY (Figure 1c).

The amygdala has been previously investigated in fear conditions, which recently has been suggested to confer a role in affecting chronic neuropathic pain [35]. More evidence indicates that the central nucleus of the amygdala receives nociceptive information from the dorsal horn of spinal cord and it can regulate the central plasticity in chronic pain [36]. After predicting the up-regulation of NPY in neuropathic pain, we then tried to investigate the potential correlation between NPY and amygdala’s effect on neuropathic pain.

The rat model of neuropathic pain-like behaviors was established by CCI of sciatic nerve. The experimental results displayed that the CCI rats showed behavioral changes, such as weakness of lateral foot walking, less weight-bearing, limping, toe closing and foot valgus. The MWT and TWL in the CCI rats were decreased and remained low in the chronic phase, and the two thresholds in the sham-operated rats were not changed. Moreover, the above pain-like behaviors were slightly ameliorated in the presence of BLA damage, along with increased MWT and TWL (Figure 1d and 1e). The results immunohistochemistry then revealed that NPY immunoreactivity was observed in sciatic nerve tissue of CCI rats, and after 7 days, CCI rats displayed significantly elevated NPY-positive cells as compared with sham-operated rats (Figure 1f and 1g).

These results suggested that NPY may contribute to the effect of amygdala nerve activity on neuropathic pain.

### NPY was highly expressed in the amygdala of rats with neuropathic pain-like behaviors

**Figure 2.**
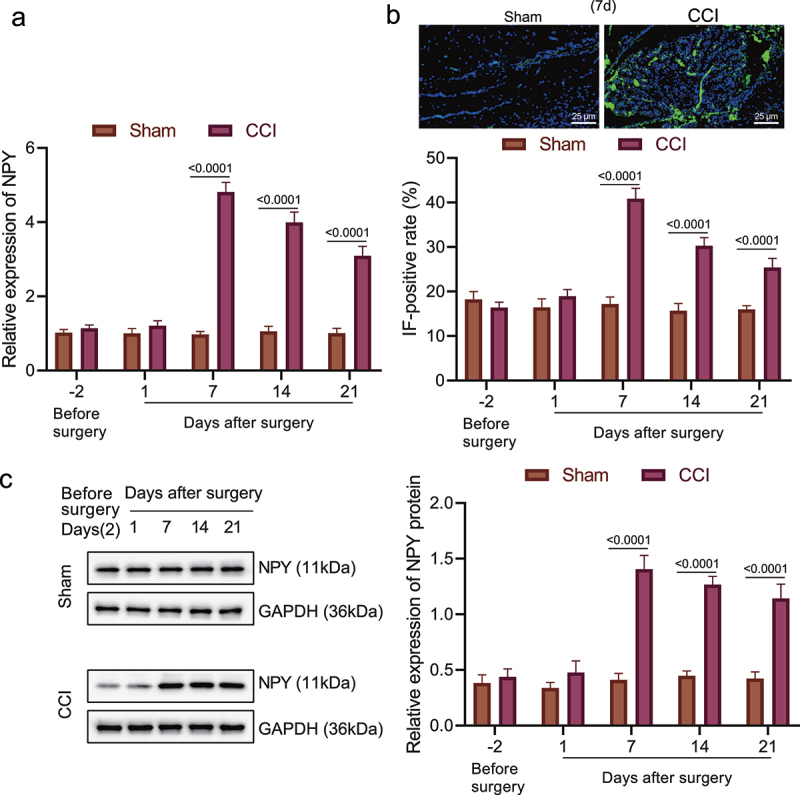
High expression of NPY occurred in the amygdala of rats with neuropathic pain-like behaviors. (a) The mRNA level of NPY in amygdala of the CCI rats and the sham-operated rats determined by RT-qPCR. (b) NPY expression in amygdala of the CCI rats and the sham-operated rats measured by immunofluorescence. (c) NPY protein level in amygdala of the CCI rats and the sham-operated rats determined by Western blot assay (n = 5).

Since the aforementioned experiments have associated NPY with the effect of amygdala on neuropathic pain, we then validated the expression of NPY in the amygdala of CCI rats. RT-qPCR was used to determine the NPY mRNA level in the CCI rats and the sham-operated rats 2 days before operation and 1, 7, 14 and 21 days after operation (Figure 2a). The NPY mRNA level increased with time and remained at a high level in the chronic phase. Then, through immunofluorescence and Western blot assays, we identified that NPY expression in amygdala of the CCI rats was the highest on the 7th day after operation (Figure 2b and 2c).

These results suggested that high expression of NPY occurred in the amygdala of rats with neuropathic pain-like behaviors was associated with NPY.

### NPY2R-activated MAPK signaling pathway might be involved in mechanism of neuropathic pain

Evidence exists reporting that NPY2R receptors, in response to stimulation, promote the development of anxiety due to its regulation in the centromedial amygdala [37]. NPY is a neurotransmitter in the spinal dorsal horn and brain tissue involved in hyperperceptual modulation in the spinal and high central nervous systems through the activation of NPY1R and NPY2R. The MAPK signaling pathway is related to NPY1R and NPY2R and is activated in neuropathologic pain [10,28,38]. It seemed that the effect of NPY in amygdala in neuropathic pain may be associated with NPY2R, we further explored the regulatory mechanism of NPY2R on MAPK pathway in neuropathic pain.

**Figure 3.**
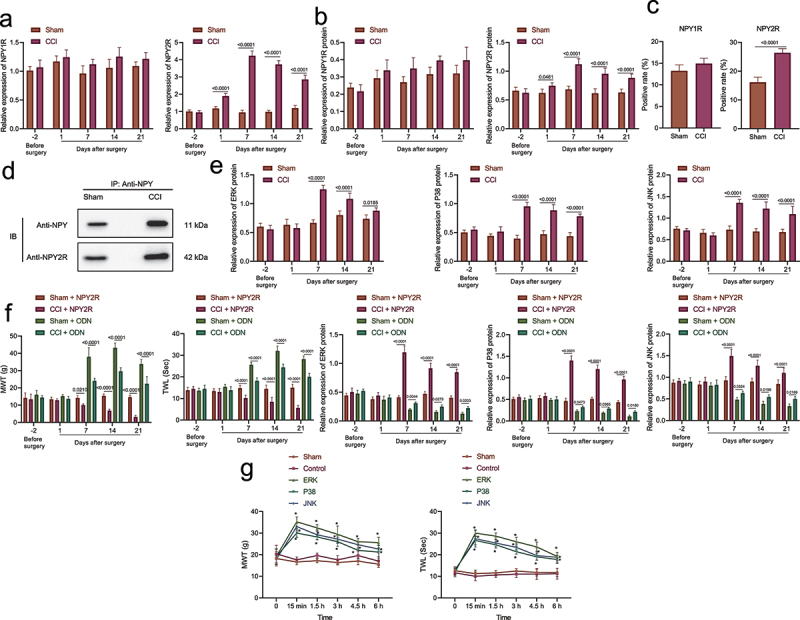
NPY2R-mediated activation of MAPK signaling pathway is involved in neuropathic pain. (a) The mRNA levels of NPY1R and NPY2R in the CCI rats and the sham-operated rats at different time points determined by RT-qPCR. (b) The protein levels of NPY1R and NPY2R in the sham-operated rats and the CCI rats and the sham-operated rats at different time points determined by Western blot assay. (c) The expression of NPY1R and NPY2R in the amygdala of CCI rats and the sham-operated rats determined by immunofluorescence staining. (d) The binding of NPY and NPY2R in the CCI rats and the sham-operated rats as observed by co-IP 21 days after operation. (e) The protein levels of ERK, p38, and JNK in the CCI rats and the sham-operated rats at different time points determined by Western blot assay. (f) The MWT and TWL (0 represents before injection) and the expression changes of MAPK signaling pathway-related proteins after injection of NPY2R agonist and NPY2R antisense ODN in the CCI rats. (g) The changes of MWT and TWL in CCI rats injected with MAPK signaling pathway antagonists (ERK antagonist, JNK antagonist, and p38 MAPK antagonist) at different time points (*n* = 5).

According to the results of RT-qPCR and Western blot assay (Figure 3a and 3b), the mRNA and protein levels of NPY2R was notably increased within 7 days after CCI modeling, but there was no significant difference in NPY1R mRNA and protein levels. Besides, no significant change was found in regard to mRNA and protein levels of NPY2R or NPY1R in the sham-operated rats. The results of immunofluorescence staining showed that NPY2R expression increased in the amygdala of CCI rats, while NPY1R expression exhibited no obvious change (Figure 3c). Moreover, Co-IP also revealed that NPY could interact with NPY2R in the CCI rats on the 21^st^ day after operation (Figure 3d). Further, expression ERK, p38 and JNK was elevated in CCI rats on the 7th day after CCI (Figure 3e).

Moreover, the injection of NPY2R agonist resulted in aggravated pain, decreased MWT and TWL, and up-regulated expression of MAPK signaling pathway-related factors in the CCI rats. However, MWT and TWL were increased after injection of ODN, and the expression of MAPK signaling pathway-related factors exhibited an obvious decline (Figure 3f). We injected MAPK signaling pathway antagonists (JNK, ERK, or p38 antagonists) into BLA of the CCI rats, and measured MWT and TWL at different time points. Based on the results, MAPK signaling pathway antagonists led to alleviated pathological pain-like behaviors as reflected by increased MWT and TWL in CCI rats, whereas no obvious difference was detected in the control rats and sham-operated rats (Figure 3g).

Overall, NPY in amygdala may activate the MAPK signaling pathway through the interaction with NPY2R, leading to the development of neuropathic pain.

### NPY2R promoted the viability and diminished the apoptosis of microglia

**Figure 4.**
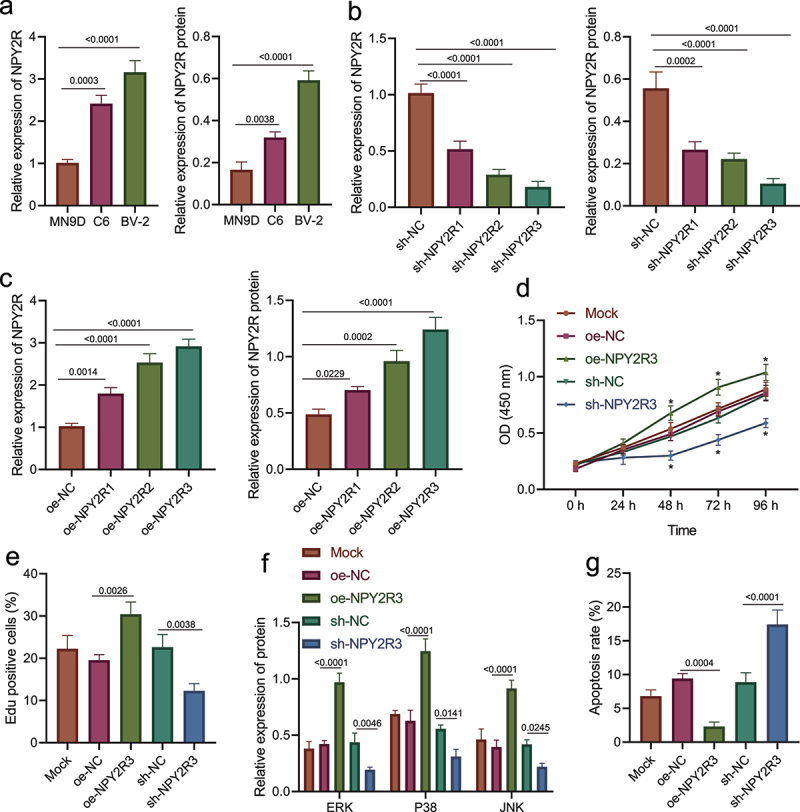
NPY2R-activated MAPK signaling pathway is associated with microglia apoptosis. (a) The mRNA and protein levels of NPY2R in MN9D, C6, and BV-2 cells determined by RT-qPCR (left) and Western blot assay (right). (b) The knockdown effect of three shRNAs targeting NPY2R in BV-2 cells determined by RT-qPCR (left) and Western blot assay (right). (c) The expression effect of three NPY2R overexpression plasmids in BV-2 cells determined by RT-qPCR (left) and Western blot assay (right). (d) The viability of BV-2 cells in response to NPY2R overexpression or knockdown as examined by CCK-8 assay. (e) The viability of BV-2 cells in response to NPY2R overexpression or knockdown as examined by EdU assay. (f) The effect of NPY2R overexpression on the expression of MAPK signaling pathway related factors (ERK, p38, and JNK) in response to NPY2R overexpression or knockdown as determined by Western blot assay. (g) The apoptosis of BV-2 cells in response to NPY2R overexpression or knockdown as as examined by flow cytometry. Cell experiments were repeated three times independently.

Furthermore, we investigated the role of NPY2R in the viability and apoptosis of microglia. The expression of NPY2R in three microglial cell lines (MN9D, C6, and BV-2) was determined by RT-qPCR and Western blot assay (Figure 4a). The results showed that NPY2R displayed the highest expression in BV-2 cell line, which was thus selected for subsequent experiments.

Then, we overexpressed or silenced NPY2R in BV-2 cells to further study its effect on microglia. Firstly, three NPY2R overexpression sequences and shRNA sequences were prepared. The results of RT-qPCR and Western blot assay revealed that sh-NPY2R-3/oe-NPY2R-3 had the most significant silencing/promotion effect on NPY2R expression in BV-2 cells (Figure 4b and 4c). Therefore, sh-NPY2R-3 and oe-NPY2R-3 were selected for subsequent experiments.

Afterward, though CCK-8 and EdU assays revealed that NPY2R overexpression augmented the viability of BV-2 cells, while NPY2R knockdown attenuated it (Figure 4d and 4e); Western blot assay showed that NPY2R overexpression promoted the expression of MAPK signaling pathway-related factors (ERK, p38, and JNK) in BV-2 cells, while NPY2R knockdown resulted in diminished expression (Figure 4f). Moreover, based on the results of flow cytometry (Figure 4g), NPY2R overexpression impeded the apoptosis of BV-2 cells, while silencing of NPY2R induced the apoptosis of BV-2 cells.

Taken together, NPY2R was capable of accelerating the viability and restricting the apoptosis of BV-2 cells.

## Discussion

At present, neuropathic pain causes a serious socioeconomic burden on a global scale with increasing incidence [39]. Unfortunately, currently available therapeutical regimens for neuropathic pain exert limited effect, making it imperative to discover novel therapies [40]. Herein, we found that NPY interacted with NPY2R to induce neuropathic pain through activating the MAPK signaling pathway.

Our initial finding in this study indicated that the NPY was highly expressed in the amygdala of rats with neuropathic pain-like behaviors and contributed to the effect of amygdala nerve activity in neuropathic pain. The amygdala has been reported for functioning in processing emotions, accountable for central plasticity in chronic pain, including neuropathic pain, greatly affected by emotional components [41]. As previously reported, NPY and its receptors are largely located in brain regions that are responsible for regulation of fear through regulation in the amygdala [42]. It has been reported that NPY has both pro-nociceptive and anti-nociceptive functions, which depends on the activation or inhibition of NPY receptor, and when Y1 receptor is activated or Y2 receptor is inhibited, it exerts analgesic effect, whereas it exhibits pain promoting effect [43]. Of note, accumulating data have unveiled that NPY and NPY2R contribute to a variety of pain-related diseases including neuropathic pain, where NPY is interacted with NPY2R [44–46]. It is also found that NPY could trigger the sensory as well as affective components in chronic neuropathic pain through inhibition of the molecular NMDAR-AC1 intracellular signaling pathway [47]. Notably, NPY has been highlighted as an important regulator in emotional disorders through binding to NPY2R [48]. As revealed by Gliosis and his colleagues, NPY, when applied to dorsal root ganglion, could contribute to induction of cold allodynia in carrageenan inflammatory pain, which is achieved by targeting NPY2R [49]. Interestingly, NPY2R was observed to be up-regulated at the first day post-spinal cord injury, where the NPYergic system was indicated to be involved in neuropathic pain induced by spinal cord injury [50]. Overall, with the support of previous researches, NPY in the amygdala induces the development of neuropathic pain by acting on its receptor NPY2R.

Furthermore, we found that the interaction between NPY and NPY2R in the amygdala could activate the MAPK signaling pathway, leading to the development of neuropathic pain. Intriguingly, the regulatory relationship between NPY/NPY2R and MAPK has been well established. For example, Schriemer *et al.* have discovered that NPY2R acts as a regulator of MAPK10 in GDNF-treated enteric neural crest cells [51]. In addition, Rosmaninho-Salgado *et al.* have revealed that activated NPYR2 could mediate the MAPK pathway, thereby inducing lipid accumulation in murine adipocytes [14]. Moreover, He *et al*. have found that the activation of NPYR2 in HEK293 cells could result in the inactivation of the MAPK/ERK signaling pathway [52]. Another study has also demonstrated that the MAPK signaling pathway is activated in neuropathic pain [28]. Besides, a large number of studies have revealed the neuropathic pain-promoting effect of activated MAPK. For instance, inactivated p38 MAPK and ERK in microglia could aid in relieving neuropathic pain-like behaviors following spinal cord injury in a rat experiment [53]. Inhibition of p38 MAPK phosphorylation by tetrapanax papyriferus and hederagenin could alleviate CCI-induced chronic neuropathic pain-like behaviors in a rat model [54]. Furthermore, the inactivated MAPK pathway in spinal microglia could lead to amelioration in neuropathic pain-like behaviors in CCI-induced rats [55]. In relation to these reports, our data also validated that NPY2R promoted the viability and diminished the apoptosis of microglia. Collectively, the role of NPY/NPY2R in neuropathic pain is associated with the activation of the MAPK signaling pathway.

## Conclusions

In summary, the evidence obtained in the current study substantiates that NPY in the amygdala can interact with NPY2R to promote the occurrence of neuropathic pain, which is achieved by activation of the MAPK signaling pathway. We hope that this finding could provide novel insights into the pain mechanism of and identify novel therapeutic targets for neuropathic pain management. Nonetheless, further studies remain to be conducted to validate the specific molecular mechanisms as well as the clinical feasibility.

## Supplementary Material

Supplemental Material

## Figures and Tables

**Table:** 

NPY	Neuropeptide Y
CCI	chronic constriction injury
LA	basolateral amygdala
MWT	mechanical withdrawal threshold
TWL	thermal withdrawal latency
MAPK	mitogen-activated protein kinase
GEO	Gene Expression Omnibus
SD	Sprague-Dawley
ODN	oligonucleotide
ERK	extracellular signal-regulated kinase
JNK	jun N-terminal kinase
RT-qPCR	Reverse transcription-quantitative polymerase chain reaction
GAPDH	Glyceraldehyde-3-phosphate dehydrogenase
TBST	tri-buffered saline Tween-20
Co-IP	Co-immunoprecipitation
DMEM	Dulbecco’s modified Eagles Medium
CCK-8	Cell counting kit-8
EdU	5-ethynyl-2-deoxyuridine
EDTA	ethylenediaminetetraacetate
FITC	fluorescein isothiocyanate conjugate
PI	propidium iodine

## Data Availability

The data supporting the findings of this study are available from the corresponding author on reasonable request.
