# Supplementary material for: Neuropeptide Y in the amygdala contributes to neuropathic pain-like behaviors in rats via the neuropeptide Y receptor type 2/mitogen-activated protein kinase axis
Source: Bioengineered. 2022 Mar 21;13(4):8101–14. doi: 10.1080/21655979.2022.2051783 (PMC9162000; doi:10.1080/21655979.2022.2051783)
Supplement: Supplemental Material [file KBIE_A_2051783_SM3800.zip › supplementary/downloadFromZipFile.pdf]

同济大学附属同济医院(上海市同济医院)

科研项目申报动物福利、伦理审查意见

<table>
<tr><td>意见号</td><td colspan="3">（同）伦审 2021-DW-SB-108</td></tr>
<tr><td>项目名称</td><td colspan="3">杏仁核中在神经性疼痛中的作用机制研究</td></tr>
<tr><td>拟申报课题类型</td><td colspan="3">2022 年度国家自然科学基金面上项目</td></tr>
<tr><td>研究单位</td><td colspan="3">同济大学附属同济医院</td></tr>
<tr><td>主要研究者</td><td colspan="3">商安全</td></tr>
<tr><td>审查日期</td><td></td><td>审查地点</td><td>动物福利伦理委员会办公室</td></tr>
<tr><td>申报文件
（需注明版本号及版本日期）</td><td colspan="3">同济大学附属同济医院国家自然科学基金青年培育项目申报书：杏仁核中在神经性疼痛中的作用机制研究</td></tr>
<tr><td>科研处意见</td><td colspan="3">申报的研究方案设计合理，具有科学和社会价值，研究设计考虑了实验动物的保护、福利和伦理，同意申报。

科研处（盖章）
日期：2021.2.6</td></tr>
<tr><td>动物福利伦理委员会意见</td><td colspan="3">申报的研究方案设计合理，充分考虑了使用最少的动物数量、采样次数，保障减少动物的痛苦，符合动物的保护、福利、伦理原则，同意申报。

动物福利伦理委员会（盖章）
日期：2021.2.18</td></tr>
</table>

扫描全能王 创建
