# Supplementary material for: Neuropeptide Y in the amygdala contributes to neuropathic pain-like behaviors in rats via the neuropeptide Y receptor type 2/mitogen-activated protein kinase axis
Source: Bioengineered. 2022 Mar 21;13(4):8101–14. doi: 10.1080/21655979.2022.2051783 (PMC9162000; doi:10.1080/21655979.2022.2051783)
Supplement: Supplemental Material [file KBIE_A_2051783_SM3800.zip › supplementary/Supplementary Tables.docx]

**Supplementary Table 1** Primer sequences for RT-qPCR

| Gene | Sequence |
| --- | --- |
| Rat-NPY | F: 5’-ATGCTAGGTAACAAGCGAATGG-3’ |
|  | R: 5’-TGTCGCAGAGCGGAGTAGTAT-3’ |
| Rat-NPY2R | F: 5’-ACCGCCATCGTTGCATTGT-3’ |
|  | R: 5’-TCAGGGAGTATTCCCGGAAGA-3’ |
| Rat-NPY1R | F: 5’-TTGCTCGTTGCGGTCATGT-3’ |
|  | R: 5’-TGGGTTGATGATTAGCTGATGC -3’ |
| Mouse-NPY2R | F: 5’-ATCTGAGAAGGAACGCGCAA-3’ |
|  | R: 5’-CCAGAGCAATGACTCTAGGAGTAG-3’ |
| Rat-GAPDH | F: 5’-GCATCTTCTTGTGCAGTGCC-3’ |
|  | R: 5’-GATGGTGATGGGTTTCCCGT-3’ |
| Mouse-GAPDH | F: 5’-CCCTTAAGAGGGATGCTGCC-3’ |
|  | R: 5’-ACTGTGCCGTTGAATTTGCC-3’ |

Note: NPY, neuropeptide Y; ERK, extracellular signalegulated kinase; JNK, Jun N-terminal kinase; GAPDH, glyceraldehyde-3-phosphate dehydrogenase; RT-qPCR, reverse transcription-quantitative polymerase chain reaction; F, forward; R, reverse

**Supplementary Table 2** Cell grouping and plasmid transfection

| Group | Plasmid |
| --- | --- |
| Mock | Blank control |
| sh-NPY2R | siRNA targeting NPY2R |
| oe-NPY2R | NPY2R overexpression plasmid |
| sh-NC | shRNA negative control |
| oe-NC | overexpression negative control plasmid |

Note: NC, negative control; NPY2R, neuropeptide Y receptor Y2
